# Factors associated with post-traumatic stress disorder and depression amongst internally displaced persons in northern Uganda

**DOI:** 10.1186/1471-244X-8-38

**Published:** 2008-05-19

**Authors:** Bayard Roberts, Kaducu Felix Ocaka, John Browne, Thomas Oyok, Egbert Sondorp

**Affiliations:** 1 Conflict and Health Programme, Health Policy Unit, Department of Public Health and Policy, London School of Hygiene and Tropical Medicine, Keppel Street, London, WC1E 7HT, UK; 2 Faculty of Medicine, Gulu University, PO Box 166, Gulu, Uganda; 3 Health Services Research Unit, Department of Public Health and Policy, London School of Hygiene and Tropical Medicine, Keppel Street, London, WC1E 7HT, UK

## Abstract

**Background:**

The 20 year war in northern Uganda between the Lord's Resistance Army and the Ugandan government has resulted in the displacement of up to 2 million people within Uganda. The purpose of the study was to measure rates of post-traumatic stress disorder (PTSD) and depression amongst these internally displaced persons (IDPs), and investigate associated demographic and trauma exposure risk factors.

**Methods:**

A cross-sectional multi-staged, random cluster survey with 1210 adult IDPs was conducted in November 2006 in Gulu and Amuru districts of northern Uganda. Levels of exposure to traumatic events and PTSD were measured using the Harvard Trauma Questionnaire (original version), and levels of depression were measured using the Hopkins Symptom Checklist-25. Multivariate logistic regression was used to analyse the association of demographic and trauma exposure variables on the outcomes of PTSD and depression.

**Results:**

Over half (54%) of the respondents met symptom criteria for PTSD, and over two thirds (67%) of respondents met symptom criteria for depression. Over half (58%) of respondents had experienced 8 or more of the 16 trauma events covered in the questionnaire. Factors strongly linked with PTSD and depression included gender, marital status, distance of displacement, experiencing ill health without medical care, experiencing rape or sexual abuse, experiencing lack of food or water, and experiencing higher rates of trauma exposure.

**Conclusion:**

This study provides evidence of exposure to traumatic events and deprivation of essential goods and services suffered by IDPs, and the resultant effect this has upon their mental health. Protection and social and psychological assistance are urgently required to help IDPs in northern Uganda re-build their lives.

## Background

There have been up to 2 million internally displaced persons (IDPs) in northern Uganda as a result of the 20 year conflict waged largely between a rebel group, the Lord's Resistance Army, and the central government and its army. Negotiations between the LRA and the Ugandan government have taken place since July 2006 but a peace settlement is yet to be signed [1]. The IDPs are based predominantly in the most conflict-affected districts of Gulu, Amuru, Kitgum and Pader which are mainly populated by the Acholi people. Up to 80% of the population in these districts are IDPs, and an estimated 85% of these IDPs live in government organised camps. The IDPs were forced to move to the camps by the Ugandan army to reportedly protect the civilians and aid the army's counter-insurgency campaign against the Lords Resistance Army [2]. The camps are characterised by chronic over-crowding, insecurity, social problems, and high rates of morbidity and mortality [3-5]. The civilian population has suffered indiscriminate killings, assaults, and the abduction of children to become fighters, forced labourers, and sex slaves [6-8]. The purpose of the study was to measure rates of post-traumatic stress disorder (PTSD) and depression amongst internally displaced persons (IDPs), and investigate associated demographic and trauma exposure risk factors.

## Methods

The study took place in November 2006 in Gulu and Amuru districts of northern Uganda (Amuru district was separated out from Gulu district in July 2006). The two districts contain an estimated 650,000 IDPs which is approximately 40% of all IDPs in Uganda. Up to 80% of the districts' population live in camps which range in size from 1,100 to almost 60,000 [9,10]. A cross-sectional survey design was used. The sampling population included adult (≥18 years old) male and female IDPs. IDPs were defined as people living in the officially recognised IDP camps in Gulu and Amuru districts. The study used the SF-8 as an outcome measure (with the findings presented elsewhere) and the sample size was calculated to detect associations of independent variables on this continuous outcome variable. The sample size required adequate power (80%) to detect conceptually important differences (0.8 standard deviation) in the health outcomes of different respondent groups within a multivariate analysis with a significance level of 5% with the size of the 'rarest' sub-group of respondents at 5% [11]. Due to the cluster sampling method used, a design effect of 2.0 was included which doubled the required sample size [12]. The expected proportion of unusable questionnaires was set at 10%. The resultant sample size required was calculated to be a minimum of 1080.

An adapted multi-stage cluster sampling method was used as random and systematic sampling methods were not feasible in the IDP camps due limited data on the population and the unsystematic layouts of the camps [12]. The first stage was to randomly select the camps from which the clusters would be chosen. The sampling frame was a list of the total population of IDPs living in all the 65 officially recognised IDP camps in Gulu and Amuru districts [10]. The data for this list was collected by the World Food Programme in August 2006 and considered to be the most accurate IDP population data available in the two districts. 32 clusters were chosen rather than the more common use of 30 clusters to reduce the design effect [13]. The 32 clusters were selected using the probability proportional to size technique. This used the World Food Programme list of the 65 officially recognised IDP camps in Gulu and Amuru districts, with a corresponding running cumulative population size for each camp. Clusters were then allocated to camps proportionally to the camp population sizes following the probability proportional to size technique to ensure self-weighting [12]. The 32 clusters were allocated to 28 IDP camps using this process. The total population living in the 28 selected camps was 452,702.

Due to the large population sizes of the selected camps, a second stage was used to randomly select administrative zones within the sampled IDP camps as second stage units to act as individual clusters. These were existing zones established by the camp authorities and were estimated to be of similar population sizes based upon discussions with camp and zone leaders, and so self-weighting was maintained. The third stage consisted of randomly choosing individuals from the selected clusters. As the clusters were already self-weighted, the same number of individuals were chosen from within each of the selected clusters. The Expanded Programme on Immunisation (EPI) method was used to randomly select households for this stage and one individual was then randomly selected from the eligible individuals within the household [13-15].

Two study staff conducted stage 2 and 3 of the sampling process through a pre-visit the day before the actual data collection. In stage 3, if the randomly selected person was not present, another adult member of the household or a neighbour were asked to inform the selected person to attend the data collection visit. The random selection process was emphasised to avoid accusations of favouritism or risk of stigmatisation against the selected person. It was advised that replacements would not be accepted and precautions were taken to reduce risk of replacements. If a household had been deserted for more than 1 month, EPI methods were followed to select another household.

A questionnaire was developed consisting of items on the demographic and socio-economic characteristics of the respondents, and existing health measurement instruments to measure trauma exposure, PTSD and depression. A slightly adapted version of the original Harvard Trauma Questionnaire (HTQ) was used to identify exposure to trauma events [16]. This consisted of 16 questions on life-time exposure to traumatic events with a 'yes/no' response. PTSD was measured using 30 questions on trauma symptoms with a 4 point severity scale and a recall period of 1 week. The first 16 items are based upon the *Diagnostic and Statistical Manual for Mental Disorders, Fourth Edition *(DSM-IV), and the remaining 14 items developed specifically for conflict-affected persons [16,17]. Mean PTSD scores ≥2.0 were considered significant for meeting symptom criteria of probable PTSD based upon the instrument standards [18]. Scores for symptoms of probable depression were measured using the 15 depression items from the Hopkins Symptoms Check List-25 (HSCL-25) [18,19]. This also has a 4 point severity scale and a recall period of 1 week. Mean depression scores ≥1.75 were considered significant for meeting symptom criteria of probable depression based upon the instrument standards [18]. The 15 depression items are consistent with the depression items in the DSM-IV [17,19]. The reliability and validity of the HTQ and HSCL-25 have been tested and proven for use with displaced persons in a number of countries [16,19-24]. The questionnaire was translated and delivered in Luo, the main language of Gulu and Amuru districts. The translation followed recommended guidelines, and involved forward and back translation, and detailed review by the study team [16,18].

A team of 15 data collectors were recruited for the survey (8 men and 7 women) who were all from the Acholi region of northern Uganda, spoke fluent Luo and English, and had experience of data collection in IDP camps in northern Uganda. Six days training was provided for the study. The data collection took place between 6 and 27 November 2006. Each interview took between 35 and 45 minutes approximately. A consent form was used to ensure informed consent and clarify that no direct benefit could be expected from participating in the study. All data collected was confidential and anonymous. Ethical approval for the study was provided by the Ugandan National Council for Science and Technology, Gulu University, and the London School of Hygiene and Tropical Medicine. As some of the questions were on traumatic experiences and mental distress, referral information for support on mental health was provided. One of the study team was a psychiatrist and one of the team leaders was a double trained Clinical Psychiatric Officer/Mental Health Nurse who could offer advice if required. Supervision and quality control were provided by the 3 members of the study team and 2 team leaders.

Two data entry clerks were used to enter the data into SPSS, version 14.0 (SPSS Inc, Chicago, USA). Each questionnaire was cross-checked by project staff and analysis conducted of the dataset to check for inconsistent data entries. Analysis was performed using STATA version 9.2 (Stata Corporation, College Park, Texas, USA) and adjusted for the clustered design. The Cronback α for internal consistency reliability was tested and estimated at 0.86 for the PTSD scale and 0.83 for the depression scale, above the generally accepted minimum threshold level of ≥0.70 for an internal reliability coefficient [25]. Multivariate logistic regression was applied to produce odds ratios (OR) of associations between independent demographic and trauma exposure variables with outcomes of PTSD and depression and adjusted to address the influence of the other significant variables. Based upon the cut off levels given in the instrument guidelines, the outcome of PTSD was dichotomised into respondents exhibiting or not exhibiting signs of PTSD (cut off ≥2.0), and the outcome of depression dichotomised into respondents exhibiting not exhibiting signs of depression (cut off ≥1.75) [18]. Continuous independent variables were also categorised for the analysis. All trauma exposure variables were included in the multivariate analyses. All demographic variables which were statistically significant (*P *< 0.05) following a univariate analysis to test for strength of association were included for the multivariate analysis. The associations in the multivariate analysis which were statistically significant (*P *< 0.05) using a backward elimination regression approach were included in the final results. Separate regression models were used for the association of independent trauma events and the cumulative events on the outcomes of PTSD and depression. Statistical interaction between independent variables was tested but none were significant (*P *< 0.05).

## Results

A total of 32 clusters were surveyed in 28 camps in Gulu and Amuru districts as planned. Table 1 presents the sampling profile of the survey. The overall response rate was 94.5%.

**Table 1:** Sampling profile

	**Gulu District**	**Amuru district**	**Total**
Camps visited	17	11	28
Absent individuals	25	19	44
Non-consenting individuals	15	7	22
Incomplete individual interviews	1	3	4
Total individual replacement	0	0	0
Completed individual interview	641	569	1210

### Sample characteristics

The sample characteristics are provided in Table 2. A greater proportion of women (60%) than men were in the sample. The mean age of respondents was 35.4 years and the main ethnic group was Acholi (91%). Over two thirds (68%) of respondents had been displaced for more than 5 years, and 41% had been displaced to 2 or more camps.

**Table 2:** Sample characteristics of Ugandan IDP respondents (N = 1210)

**Characteristics**	**Number (%)**
Number of women	727 (60.1)

Age, mean	35.3 years

Religion	
Catholic	927 (76.6)
Protestant	167 (13.8)
Born again Christian	101 (8.4)
other	15 (1.2)

Ethnicity	
Acholi	1,098 (90.7)
Lango	88 (7.3)
Other	24 (2.0)

Marital status	
married/co-habiting	926 (76.5)
single	72 (6.0)
separated*	212 (17.5)

Education level	
never attended school	380 (31.4)
completed primary school	711 (58.8)
completed secondary school	119 (9.8)

Personal income of previous month §	
<25,000 UGS	732 (60.5)
25,000–50,000 UGS	230 (19.0)
>50,000 UGS	238 (19.7)
refused to answer	10 (0.8)

Period since left village	
Less than 12 months	20 (1.7)
1 – 2 years	69 (5.7)
3 – 5 years	277 (22.9)
5 – 10 years	479 (39.6)
More than 10 years	345 (28.5)
Don't Know	20 (1.7)

Number of camps lived in	
1 camp	711 (58.8)
2 camps	386 (31.90)
3 or more camps	113 (9.3)

Abbreviations: UGS, Ugandan shillings

§1 USD = 1800 UGS at time of survey

* Separated: divorced/separated, widowed, forcibly separated

### Exposure to trauma

**Figure 1:**
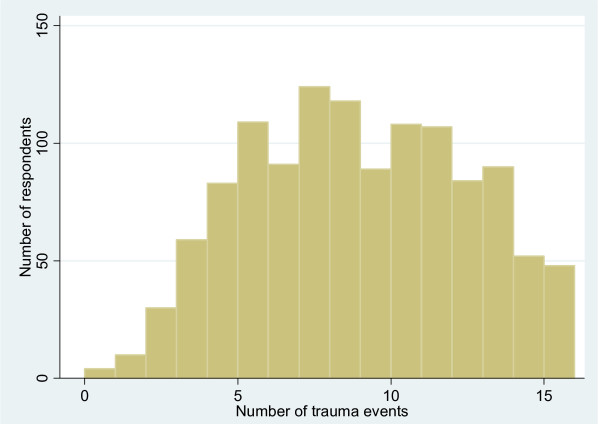
Distribution of trauma exposure (N = 1210).

The results on exposure to traumatic events are given in Table 3. Three quarters (75%) of respondents had witnessed or experienced the murder of a family or friend. Nearly two thirds (64%) of respondents had witnessed the murder of a stranger or strangers. More than half (56%) reported having been beaten or tortured. More than 40% reported having been kidnapped and 14% reported having been raped or sexually abused. Over half (58%) of respondents had experienced 8 or more of the 16 trauma events covered in the questionnaire. The distribution of the exposure to traumatic events is shown in Figure 1.

**Table 3:** Exposure to Traumatic Events (N = 1210)

**Variables**	**Number of persons experiencing event**			**Total (%) [95% CI]**
	Before living in camp	When living in camp	before and when living in camp	
Trauma event experienced				
lack of food or water	77	940	71	1088 (89.9) [87.9–91.7]
lack of housing or shelter	76	815	44	935 (77.3) [73.5–80.7]
unnatural death of family/friend	524	298	93	915 (75.6) [73.7–77.5]
murder of family member/friend	578	236	91	905 (74.8) [71.4–78.0]
being close to, but escaping, death	549	249	47	845 (69.8) [67.0–72.6]
ill health without medical care	176	554	57	787 (65.0) [61.8–68.2]
witnessing murder of stranger(s)	390	308	80	778 (64.3) [61.4–67.1]
tortured or beaten	469	181	29	679 (56.1) [52.4–59.8]
forced separation from family	392	133	25	550 (45.5) [42.6–48.3]
being abducted or kidnapped	370	133	21	524 (43.3) [39.8–46.9]
made to accept ideas against will	147	279	57	483 (39.9) [36.6–43.5]
serious injury	282	178	14	474 (39.2) [36.1–42.4]
forced isolation from other people	293	149	10	452 (37.4) [34.3–40.6]
being in a war fighting situation	235	85	10	330 (27.3) [24.0–30.9]
imprisonment against your will	187	101	9	297 (24.5) [22.0–27.3]
rape or sexual abuse	78	83	10	171 (14.1) [11.8–16.8]

Cumulative trauma events recorded				
0–3 trauma events				103 (8.5) [7.2–10.0]
4–7 trauma events				408 (33.7) [30.8–36.8]
8–11 trauma events				425 (35.1) [32.7–37.6]
12–16 trauma events				274 (22.6) [20.0–25.6]

Abbreviations: CI, confidence interval.

There were significant variations between women and men in exposure to a number of the traumatic events. 47% [95% CI 41.99–51.61] of women and 70% [95% CI 65.01–74.89] of men had experienced being beaten or tortured. 18% [95% CI 15.35–21.97] of women and 8% [95% CI 5.37–10.82] of men had been raped or sexually abused. 31% [95% CI 26.8–35.14] of women and 62% [95% CI 56.34–67.56] of men had been abducted. 13% [95% CI 11.00–16.10] of women and 41% [95% CI 36.6–46.39] of men had been imprisoned. 23% [95% CI 19.26–26.54] of women and 34% [95% CI 28.79–39.97] of men had been involved in combat. 49% [95% CI 44.47–52.93] of women and 71% [95% CI 67.19–75.32] of men had experienced 8 or more traumatic events.

Deprivation by IDPs of material and social goods is evidenced by the fact that 90% respondents had experienced lack of food or water, over two thirds (65%) had been ill without medical care, and over three quarters (77%) had lacked housing or shelter. Over half of the traumatic events listed in Table 3 had occurred whilst the participants were living in a camp. 93% did not feel safe in the camp in which they lived. The main reasons cited for this perceived lack of safety were lack of food (62%), fear of disease outbreak (61%), and insecurity and fear of armed forces (54%).

### Prevalence of PTSD and depression

**Figure 2:**
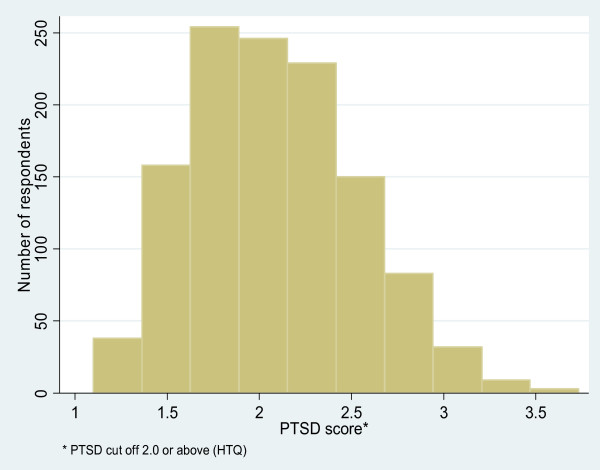
Distribution of PTSD scores (N = 1210).

**Figure 3:**
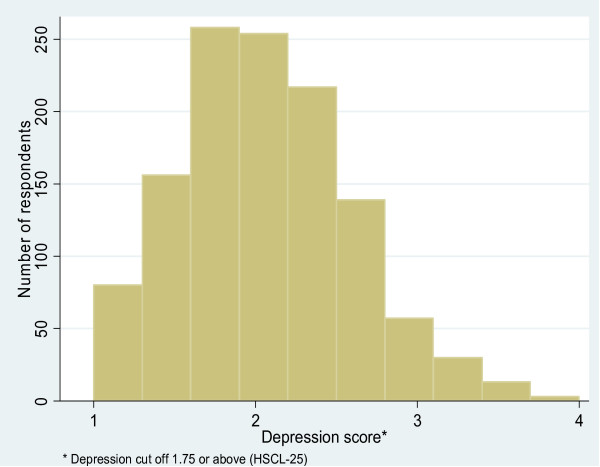
Distribution of depression scores (N = 1210).

More than half (54%) of respondents met symptom criteria for PTSD (Table 4). 60% of women met symptom criteria for PTSD. Over two thirds (67%) of respondents met symptom criteria for depression. 78% of women met symptom criteria for PTSD. The distribution of the scores for PTSD and depression are shown in Figures 2 and 3.

**Table 4:** Prevalence rate of PTSD and depression

**Variable**	**Total (%) [95% CI]**
Symptoms of PTSD	
men (n = 483)	220 (45.6) [39.9–51.3]
women (n = 727)	437 (60.1) [55.8–64.2]
total (n = 1210)	657 (54.3) [50.4–58.1]

Symptoms of depression	
men (n = 483)	248 (51.4) [45.8–56.9]
women (n = 727)	567 (78.0) [74.1–81.4]
total (n = 1210)	815 (67.4) [63.5–71.0]

Abbreviations: CI, confidence interval; PTSD, post-traumatic stress disorder.

Symptoms of PTSD (HTQ PTSD Score = ≥2.0) and depression (HSCL score = ≥1.75)

All results *P *< 0.05

### Multivariate analysis

Table 5 shows the statistically significant (*P *< 0.05) adjusted odds ratio results of the multivariate logistic regression analysis on the association of key demographic and trauma exposure variables on the outcomes of PTSD (cut off ≥2.0) and depression (cut off ≥1.75). These show a strong association of gender on mental distress, with women twice as likely as men to be exhibit symptoms of PTSD (OR 2.06 [95% CI 1.49–2.84]) and over 4 times as likely as men to exhibit symptoms of depression (OR 4.32 [95% CI 2.80–6.66]). Other demographic variables associated with mental distress include the marital status of respondents, with respondents who were no longer married (divorced/separated, widowed, or forced separation) more likely than married respondents to exhibit symptoms of PTSD (OR 1.80 [95% CI 1.25–2.59]) and depression (OR 2.41 [95% CI 1.36–4.27]). The distance of displacement (camp >5 miles from home village) was also associated with depression (OR 1.47 [95% CI 1.16–1.85]).

**Table 5:** Multivariate analysis of demographic and individual trauma exposure variables associated with PTSD and depression

**Variable**	**PTSD (N = 657)**		**Depression (N = 815)**	
	OR [95% CI]	*P *value	OR [95% CI]	*P *value
Demographic Variables				
Female	2.06 [1.49–2.84]	<0.01	4.32 [2.80–6.66]	<0.01
no longer married*	1.80 [1.25–2.59]	<0.01	2.41 [1.36–4.27]	<0.01
displaced > 5 miles from home	NA	NA	1.47 [1.16–1.85]	<0.01

Trauma exposure variable				
ill health without medical care	1.95 [1.52–2.51]	<0.01	1.97 [1.50–2.58]	<0.01
rape or sexual abuse	1.67 [1.01–2.75]	0.045	NA	NA
lack of food or water	1.55 [1.00–2.39]	0.048	1.64 [1.05–2.58]	0.03
unnatural death of family/friend	NA	NA	1.54 [1.10–2.16]	0.01
being tortured or beaten	1.42 [1.09–1.84]	0.01	1.41 [1.04–1.92]	0.03
being made to accept ideas/brainwashing	1.39 [1.03–1.88]	0.03	NA	NA
witnessing murder of stranger or strangers	1.38 [1.02–1.86]	0.04	NA	NA
serious injury	NA	NA	1.38 [1.09–1.74]	<0.01

Abbreviations: CI, confidence interval; PTSD, post-traumatic stress disorder; OR, odds ratio (adjusted); NA, not applicable or was not statistically significant (P > 0.05).

Multivariate analysis for demographic variables in table, and all individual trauma exposure variables shown in Table 3. Only statistically significant demographic variables (*P *< 0.05) following univariate analysis included in multivariate analysis.

Only statistically significant (P < 0.05) adjusted odds ratios from multivariate analysis presented.

* No longer married includes divorced/separated, widowed, forcibly separated.

Symptoms of PTSD (HTQ PTSD Score = ≥2.0) and depression (HSCL score = ≥1.75)

The individual trauma exposures with the strongest associations with PTSD were ill health without medical care (OR 1.95 [95% CI 1.52–2.51]); rape or sexual abuse (OR 1.67 [95% CI 1.01–2.75]); and lack of food or water (OR 1.55 [95% CI 1.00–2.39]). The individual trauma exposures with the strongest associations with depression were ill health without medical care (OR 1.97 [95% CI 1.50–2.58]); lack of food or water (OR 1.64 [95% CI 1.05–2.58]; and unnatural death of family/friend (OR 1.54 [95% CI 1.10–2.16]).

Table 6 shows the statistically significant (*P *< 0.05) adjusted odds ratio results of the multivariate logistic regression analysis on the association of cumulative trauma exposure on the outcomes of PTSD (cut off ≥2.0) and depression (cut off ≥1.75). A higher frequency of exposure to traumatic events (experienced 12 or more of the 16 trauma events included in the questionnaire) was associated with exhibiting symptoms of PTSD (OR 6.5 [95% CI 3.7–11.3]) and depression (OR 5.8 [95% CI 3.5–9.7]).

**Table 6:** Multivariate analysis of demographic variables and cumulative trauma variables associated with PTSD and depression

**Variable**	**PTSD (N = 657)**		**Depression (N = 815)**	
	OR [95% CI]	*P *value	OR [95% CI]	*P *value
Demographic Variables				
female	2.20 [1.67–2.90]	<0.01	4.12 [2.91–5.84]	<0.01
no longer married*	1.80 [1.26–2.59]	<0.01	2.62 [1.46–4.69]	<0.01
displaced > 5 miles from home	NA	NA	1.46 [1.18–1.82]	<0.01

Cumulative trauma events				
0–3 trauma events	ref		ref	
4–7 trauma events	2.43 [1.3–4.4]	<0.01	2.31 [1.4–3.8]	<0.01
8–11 trauma events	4.62 [2.7–7.8]	<0.01	5.07 [3.1–8.4]	<0.01
12–16 trauma events	6.51 [3.7–11.3]	<0.01	5.84 [3.5–9.7]	<0.01

Abbreviations: CI, confidence interval; PTSD, post-traumatic stress disorder; OR, odds ratio (adjusted); NA, not applicable as not statistically significant (P > 0.05).

* No longer married includes divorced/separated, widowed, forcibly separated.

Multivariate analysis for demographic variables in table, and cumulative trauma event categories. Only statistically significant demographic variables (*P *< 0.05) following univariate analysis included in multivariate analysis.

Only statistically significant (P < 0.05) adjusted odds ratios from multivariate analysis presented.

Symptoms of PTSD (HTQ PTSD Score = ≥2.0) and depression (HSCL score = ≥1.75)

## Discussion

This study provides evidence of extremely high exposure to traumatic events suffered by civilians in Gulu and Amuru districts of northern Uganda and indicates widespread human rights abuses in northern Uganda, corroborating other findings on trauma exposure in northern Uganda [26]. 43% of respondents reporting having been abducted or kidnapped. Three quarters of respondents witnessed or experienced the murder of a family or friend, and over half of respondents reported having been beaten or tortured. Almost one in seven respondents had experienced rape and sexual abuse. These rates of rape and sexual abuse are substantially higher than reported in other studies of displaced persons using similar methodologies [24,27-29]. Evidence on sexual abuse of men is rare in conflict-affected populations, but in this study 8% of male respondents reported having been raped or sexually abused. Deprivation of essential goods and services by IDPs is demonstrated by the fact that 90% of respondents had experienced lack of food or water, almost two thirds had experienced ill health without access to medical care, and over three quarters had lacked housing or shelter. Over half of respondents had experienced 8 or more of the 16 trauma events covered in the questionnaire. The IDP camps were established by the Ugandan government to protect the civilians population but over half of the traumatic events listed in Table 3 had occurred while the respondents were living in a camp.

This study reveals extremely high levels of psychiatric morbidity amongst the IDP population in Gulu and Amuru districts. 54% of respondents met symptom criteria for PTSD and 67% of respondents met symptom criteria for depression. These results compare with rates for symptoms of PTSD and depression in Gulu district of 71% and 31% recorded in a previous study [26]. The study results of depression compare with rates of 26% in Adjumani district which has also been affected by the war in northern Uganda but less so than Gulu and Amuru districts, and rates of 17% in Bugiri district in the East of Uganda which has not been affected by the war in the North [30].

The levels of PTSD and depression recorded in this study are amongst the highest recorded globally using similar methodologies amongst displaced and conflict-affected populations. Rates of PTSD and depression amongst Guatemalan refugees in Mexico were recorded at 11.8% and 38% respectively [24]. Amongst Karenni refugees living in the Thai-Burma border, 4.6% and 41.8% of respondents met criteria for PTSD and depression [27]. A survey of Bosnian refugees in Croatia diagnosed PTSD and depression in 5.6% and 18.6% of respondents. In Afghanistan, rates of PTSD have varied between 20.4% to 42% and rates for depression from 38.5% to 68% [29,31].

The study found a number of significant associations of independent variables on outcomes of PTSD and depression. Women are at particularly high risk of poor mental health, along with people that are no longer married, as recorded in other studies on mental health of displaced populations [24,26-29,32]. Traumatic events with significant associations with PTSD and depression included rape or sexual abuse, unnatural death of family/friend, murder of stranger or strangers, and being tortured or beaten. The dose-response relationship between exposure to traumatic events and PTSD and depression is also consistent with other studies of displaced population [24,27,28,33,34]. This study also showed that the absence of basic social goods and services such as food, water and health care had a significant association with outcomes of PTSD and depression. The association between absence of food and poor mental health is reflected in some other studies of displaced populations [24,27]. This study also showed that while men reported higher exposure to traumatic events than women, men reported lower levels of mental distress. It has been previously suggested that women may be at higher risk of mental distress because of the psychological consequences of rape, the violent loss of partner and children, and of becoming a single parent or widow [35]. Pre-traumatic and post-traumatic factors have also been shown to influence levels of PTSD [36,37]. Further investigation on resilience amongst persons exposed to trauma and not exhibiting signs of PTSD is also required.

### Limitations

This study has a number of limitations. Firstly, the cross-sectional design means the findings cannot be generalised across northern Uganda, and only associations can be described between variables rather than attributing causation. Secondly, the study cannot highlight individual camps with particular needs because precision is too low due to the cluster survey design. Thirdly, the study was unable to consistently match the gender of interviewer and respondents. As a result, there may have been underreporting of certain sensitive traumatic events. Fourthly, IDPs not living in officially registered camps (for example, those hosted by relatives or friends or living in unregistered camps in Gulu Municipality) were not included in the study. These IDPs represent approximately 21% of the entire IDP population of Gulu and Amuru districts [4]. It is difficult to assess how the study health outcomes may vary between IDPs living in registered camps and those who are not. However, mortality outcomes between the two groups appear broadly similar [4]. Lastly, the validity of measuring mental health outcomes like PTSD in different cultural settings has been questioned [38,39]. However, the HTQ and HSCL-25 used in this study have been specifically developed for conflict-affected populations and have been widely used and validated in Asia, Africa, Latin America and Europe [16,19-24,26,27,40]. The instruments also had high internal reliability levels in this study using the Cronbach α. However, an in-depth validation of the HTQ and HSCL instruments, including locally-developed cut off points, would make an important contribution to the understanding of mental health amongst IDPs in northern Uganda.

## Conclusion

This study provides evidence to indicate vulnerability to trauma exposure and deprivation of essential goods and services suffered by IDPs in northern Uganda, and the resultant effects this has upon their mental health. Under international law, the primary responsibility for providing humanitarian protection and assistance to IDPs lies with the national authorities. However, the international community also has a right to offer support to IDPs but this right has not always been adequately fulfilled [41-43]. The Ugandan government and the international community need to strengthen comprehensive protection and social support for IDPs in the camps of northern Uganda to help reduce mental distress and as a preventative measure to reduce further exposure to trauma. Some direct interventions to reduce mental distress have shown some impact in northern Uganda, but further investigation on the effectiveness and feasibility of these interventions is required given the size of the population affected [44]. A meaningful resolution to the conflict in northern Uganda would facilitate a return by IDPs back to their homes, support a healing process, and help IDPs re-build their lives.

## Abbreviations

CI: Confidence Interval; HTQ: Harvard Trauma Questionnaire; HSCL-25: Hopkins Symptoms Check List-25; IDP: Internally Displaced Person; OR: Odds Ratio; PTSD: Post-traumatic stress disorder.

## Competing interests

The authors declare that they have no competing interests.

## Authors' contributions

BR led the study concept and design, data collection, data analysis, and drafting of the manuscript. KFO participated in developing the study concept and design, data collection, review of data analysis, and review of the manuscript. JB participated in developing the study concept and design, review of data analysis, and review of the manuscript. TO participated in developing the study concept and design, data collection, and review of the manuscript. ES participated in developing the study concept and design, and reviewing the manuscript. All authors read and approved the final manuscript.

## Pre-publication history

The pre-publication history for this paper can be accessed here:


